# Supplementary material for: Correlation between Statin Solubility and Mortality in Patients on Chronic Hemodialysis
Source: Diagnostics (Basel). 2023 Oct 23;13(20):3290. doi: 10.3390/diagnostics13203290 (PMC10605997; doi:10.3390/diagnostics13203290)
Supplement: Supplementary file 1 [file diagnostics-13-03290-s001.zip › diagnostics-2635114-supplementary materials.pdf]

Supplementary materials

Table S1. Medication types and Health Insurance Review and Assessment Service codes

Table S2. The ICD–10 codes were used in Charlson Comorbidity Index

Figure S1. Forest plots of the HR and 95% CI according to subgroups

Table S1. Medication types and Health Insurance Review and Assessment Service codes

Medications	Codes
**Acepril**	104201ATB, 104202ATB
**Amlodipine**	495901ATB, 459802ACH, 483201ATB, 486501ATB, 107601ATB, 107601ATD, 459801ACH, 459801ATB, 459901ATB, 464601ATB, 470801ATB, 476201ATB, 479701ATB, 483202ATB, 486502ATB, 107602ATB, 107602ATD, 470802ATB
**Amlodipine+Atorvastatin**	614500ATB, 472300ATB472400ATB472500ATB518900ATB
**Amlodipine+Losartan+Chlorthalidone**	662800ATB, 662900ATB, 663000ATB
**Amlodipine+Losartan+Rosuvastatin**	663900ATB, 664000ATB, 664100ATB, 664200ATB, 664300ATB, 664400ATB
**Amlodipine+Olmesartan+Rosuvastatin**	677300ATB, 677400ATB, 677500ATB, 677600ATB
**Amlodipine+Rosuvastatin**	673900ATB, 674000ATB, 674100ATB
**Amlodipine+Rosuvastatin+Telmisartan**	671200ATB, 671300ATB, 671400ATB, 671500ATB, 677000ATB, 677100ATB, 671600ATB, 671700ATB
**Amlodipine+Telmisartan+Hydrochlorothiazide**	663500ATB, 663600ATB, 663700ATB, 663800ATB
**Amosulalol**	107901ATB, 107902ATB
**Arotinolol**	110202ATB, 110201ATB
**Atenolol**	483102ATB, 111402ATB, 483101ATB, 111403ATB, 111401ATB
**Atenolol+Chlorthalidone**	262100ATB, 460200ATB
**Azilsartan**	662401ATB, 662403ATB, 662402ATB
**Azilsartan+Chlorthalidone**	673500ATB, 673600ATB
**Barnidipine**	114003ACH, 114001ACH, 114002ACH
**Benidipine**	115101ATB, 115102ATB, 115104ATB, 115103ATB
**Betaxolol**	116801ATB, 116803ATB
**Bevantolol**	117002ATB, 117001ATB
**Bisoprolol**	117904ATB, 117903ATB, 117902ATB, 117901ATB
**Bisoprolol+Hydrochlorothiazide**	469800ATB, 470000ATB, 469900ATB

**Candesartan**	122601ATB, 122602ATB, 122603ATB
**Candesartan+Amlodipine**	652900ATB, 653000ATB, 653100ATB
**Candesartan+Hydrochlorothiazide**	423700ATB
**Candesartan+Rosuvastatin**	661800ATB, 661900ATB, 673700ATB, 662000ATB, 662100ATB
**Captopril**	122901ATB, 122902ATB, 122903ATB
**Captopril+Hydrochlorothiazide**	262200ATB, 262300ATB
**Carteolol**	124801ATB
**Carvedilol**	125005ATB, 125003ATB, 662201ATB, 125008ACR, 125001ATB, 662202ATB, 125007ACR, 125002ATB, 125006ACR, 125004ACR
**Celiprolol**	129101ATB
**Cilazapril**	133001ATB, 133002ATB, 133003ATB
**Cilnidipine**	133102ATB, 133101ATB
**Clonidine**	136505ATR
**Diltiazem**	145706ATB, 145707ACR, 145707ATR, 145703ACR, 145706ATR, 145707ATB
**Doxazocin**	149101ATB, 149102ATB, 149104ATR, 149103ATB
**Efonidipine**	441202ATB, 441201ATB
**Enalapril**	151603ATB, 151601ATB
**Enalapril+Hydrochlorothiazide**	453700ATB, 440300ATB
**Eprosartan**	429201ATB
**Eprosartan+Hydrochlorothiazide**	460500ATB
**Felodipine**	157503ATR, 157501ATR
**Felodipine+Metoprolol**	262400ATR
**Fimasartan**	515203ATB, 515201ATB, 515202ATB
**Fimasartan+Amlodipine**	651900ATB, 652000ATB, 652700ATB, 652100ATB
**Fimasartan+Hydrochlorothiazide**	522000ATB, 526800ATB
**Fimasartan+Rosuvastatin**	655000ATB, 654900ATB, 654800ATB, 654700ATB, 654600ATB
**Fosinopril**	163501ATB, 163502ATB

**Hydralazine**	170701ATB
**Imidapril**	173402ATB, 173401ATB
**Irbesartan**	177301ATB, 177303ATB
**Irbesartan+Atorvastatin**	524000ATB, 524100ATB, 527100ATB, 527000ATB
**Irbesartan+Hydrochlorothiazide**	385700ATB, 385800ATB, 553800ATB
**Lacidipine**	180301ATB, 180302ATB, 180303ATB
**Lercanidipine**	182001ATB, 182002ATB
**Lisinopril**	184501ATB
**Lisinopril+Hydrochlorothiazide**	499200ATB, 499300ATB
**Losartan**	185701ATB, 185702ATB
**Losartan+Amlodipine**	503000ATB, 637400ATB, 513900ATB, 637500ATB, 502700ATB, 637600ATB
**Losartan+Hydrochlorothiazide**	262500ATB, 486900ATB, 378900ATB
**Manidipine**	188001ATB, 188002ATB
**Metoprolol**	194003ATR, 193802ATB, 262400ATR
**Metoprolol+Hydrochlorothiazide**	262600ATB
**Metoprolol+felodipine**	262400ATR
**Minoxidil**	196102ATB
**Nadolol**	198301ATB
**Nebivolol**	489501ATB, 489502ATB, 489503ATB
**Nicardipine**	201003ACR, 201002ATB
**Nifedipine**	201407ACS, 201405ATR, 528201ATR, 201409ATR, 528202ATR, 201401ACS, 201401ATB, 201408ATR
**Nimodipine**	201901ATB, 356202ATR, 356203ATR, 356201ATB, 356202ATB
**Nisoldipine**	356202ATR
**Olmesartan**	468502ATB, 468501ATB, 468503ATB, 520902ATB, 520901ATB

**Olmesartan+Amlodipine**	547800ATB, 632800ATB, 500500ATB, 547700ATB, 629500ATB, 631300ATB, 500600ATB, 547900ATB, 632900ATB, 547600ATB, 548000ATB, 582200ATB, 629600ATB, 633000ATB, 547500ATB, 582400ATB, 629400ATB
**Olmesartan+Amlodipine+Hydrochlorothiazide**	519800ATB, 519700ATB, 520100ATB, 520000ATB, 519900ATB
**Olmesartan+Hydrochlorothiazide**	513600ATB, 489100ATB
**Olmesartan+Rosuvastatin**	644200ATB, 644100ATB, 526900ATB, 526300ATB, 526400ATB, 653200ATB, 526500ATB
**Perindopril**	211301ATB, 501601ATB, 211302ATB, 501602ATB
**Perindopril+Indapamide**	556200ATB
**Propranolol**	219901ATB, 219904ATB, 219906ACR, 219905ACR
**Quinapril**	221901ATB
**Ramipril**	222401ATB, 222402ATB, 222404ATB
**Ramipril+Felodipine**	447100ATB, 447200ATB
**Ramipril+Hydrochlorothiazide**	448600ATB, 448700ATB
**Telmisartan**	378801ATB, 378802ATB, 378803ATB
**Telmisartan+Amlodipine**	521200ATB, 511600ATB, 521300ATB, 511700ATB, 521400ATB, 511500ATB, 644800ATB, 623100ATB
**Telmisartan+Hydrochlorothiazide**	443200ATB, 443300ATB, 502600ATB
**Telmisartan+Rosuvastatin**	631600ATB, 629900ATB, 630000ATB, 631700ATB, 630100ATB, 630200ATB
**Temocapril**	235002ATB
**Terazosin**	235501ATB, 235502ATB, 235503ATB, 616501ATB
**Valsartan**	247103ATB, 247101ATB, 247102ATB, 247104ATB
**Valsartan+Amlodipine**	522600ATB, 492900ATB, 522900ATB, 523200ATB, 522700ATB, 492800ATB, 522800ATB, 523000ATB, 523300ATB, 495800ATB, 523100ATB, 523400ATB
**Valsartan+Hydrochlorothiazide**	356400ATB, 442600ATB
**Valsartan+Lercanidipine**	522200ATB, 522300ATB, 522400ATB
**Valsartan+Pitavastatin**	635000ATB, 635200ATB, 634900ATB, 635100ATB

**Valsartan+Rosuvastatin**	629700ATB, 525000ATB, 525200ATB, 629800ATB, 525100ATB, 525300ATB
**Valsartan+Sacubitril**	651401ATB, 651402ATB, 651403ATB
**Verapamil**	247606ATB, 247607ATB, 247603ATR, 247605ATR, 247601ACR
**Zofenopril**	510401ATB, 510402ATB, 510403ATB
**Atorvastatin+Amlodipine**	472300ATB, 472400ATB
**Atorvastatin+Ezetimibe**	633800ATB, 633900ATB, 634800ATB
**Pitavastatin+Fenofibrate**	679300ACH
**Rosuvastatin+Ezetimibe**	640700ATB, 640800ATB, 640900ATB
**Rosuvastatin+Ezetimibe+Telmisartan**	671400ATB, 671500ATB, 671700ATB
**Aspirin**	110701ATB, 110702ATB, 110801ATB, 110802ATB, 111001ACE, 111001ATB, 111001ATE, 111002ATE, 111003ACE, 111003ATE
**Clopidogrel**	133201ACR, 133201ATB, 133201ATR, 133202ATB, 133203ATR, 506100ATB
**Cilostazol**	136901ATB, 492501ATB, 495201ATB, 498801ATB, 501501ATB
**Ticlopidine**	498900ATB, 239201ATB, 239202ATB
**Aspirin+Bethocarbamol**	256800ATB
**Aspirin+Clopidogrel**	517900ACH, 517900ACE, 517900ATE, 667500ACE
**Aspirin+Dipyridamole**	489700ACR
**Atorvastatin 10mg**	111501ATB, 502201ATB, 633800ATB, 472300ATB, 524000ATB, 527100ATB, 614500ATB, 671800ATR, 671900ATR, 673800ATR
**Atorvastatin 20mg**	111502ATB, 502202ATB, 633900ATB, 472400ATB, 518900ATB, 524100ATB, 527000ATB, 672000ATR, 672100ATR
**Atorvastatin 40mg**	111503ATB, 502203ATB, 634800ATB, 472500ATB,
**Atorvastatin 80mg**	111504ATB, 502204ATB
**Fluvastatin 20mg**	162401ACH
**Fluvastatin 40mg**	162402ACH
**Fluvastatin 80mg**	162403ATR
**Lovastatin 20mg**	185801ATB

**Pitavastatin 1mg**	470902ATB
**Pitavastatin 2mg**	470901ATB, 634900ATB, 635000ATB
**Pitavastatin 4mg**	470903ATB, 635100ATB, 635200ATB
**Pravastatin 5mg**	216602ATB
**Pravastatin 10mg**	216601ATB
**Pravastatin 20mg**	216603ATB
**Pravastatin 40mg**	216604ATB, 519300ACH
**Rosuvastatin 5mg**	454003ATB, 640700ATB, 663400ACS, 526900ATB, 629700ATB, 629800ATB, 631600ATB, 631700ATB, 644200ATB, 654800ATB, 655000ATB, 661800ATB, 663900ATB, 664200ATB, 671200ATB, 671300ATB, 664600ATB, 631600ATB, 631700ATB, 673700ATB, 673900ATB, 672500ATR, 672600ATR,
**Rosuvastatin 10mg**	454001ATB, 640800ATB, 525000ATB, 525100ATB, 526300ATB, 629900ATB, 630100ATB, 644100ATB, 653200ATB, 654700ATB, 654900ATB, 661900ATB, 662000ATB, 664000ATB, 664300ATB, 671400ATB, 671500ATB, 671600ATB, 664700ATB, 674000ATB, 672700ATR, 672800ATR
**Rosuvastatin 20mg**	454002ATB, 640900ATB, 525200ATB, 525300ATB, 526400ATB, 526500ATB, 630000ATB, 630200ATB, 654600ATB, 622100ATB, 664100ATB, 664400ATB, 671700ATB, 664800ATB, 674100ATB, 672900ATR, 673000ATR
**Simvastatin 5mg**	227806ATB
**Simvastatin 10mg**	471000ATB, 227803ATB,
**Simvastatin 20mg**	227801ATB, 227801ATR, 471100ATB, 631400ATB,
**Simvastatin 40mg**	227802ATB, 507800ATB, 631500ATB
**Simvastatin 80mg**	227805ATB

Table S2. The ICD–10 codes were used in Charlson Comorbidity Index

Comorbidities	Codes	Score
Myocardial infarction	I21, I22, I252	1
Congestive heart failure	I43, I50, I099, I110, I130, I132, I255, I420, I425-I429, P290	1
Peripheral vascular disease	I70, I71, I731, I738, I739, I771, I790, I792, K551, K558, K559, Z958, Z959	1
Cerebrovascular disease	G45, G46, I60-69, H340	1
Dementia	F00-03, G30, F051, G311	1
Chronic pulmonary disease	J40-47, J60-67, I278-279, J701, J703, J684	1
Rheumatologic disease	M05–06, M32–34, M315, M351, M353, M360	1
Peptic ulcer disease	K25–28	1
Mild liver disease	B18, K73, 74, K700-703, K709, K713-715, K717, K760, K762-764, K768–769, Z944	1
DM without complication	E100-101, E106, E108-111, E116, E118-121, E126, E128-131, E136, E138-141, E146, E148-149	1
DM with complication	E102-105, E107, E112-115, E117, E122-125, E127, E132-135, E137, E142-145, E147	2
Hemiplegia or paraplegia	G81-82, G041, G114, G800, G830-834, G839	2
Any malignancy	C00-26, C30-C34, C37-41, C43, C45-58, C60-6, C81-88, C90-97	2
Moderate to severe liver disease	I850, I859, I864, I982, K704, K711, K721, K729, K765-767	3
Metastatic tumor	C77-80	6
AIDS/HIV	B20-22, B24.	6

Abbreviations: ICD–10, International Classification of Diseases, 10th revision, Clinical Modification; DM, diabetes mellitus; AIDS/HIV, acquired immune deficiency syndrome/human immunodeficiency virus

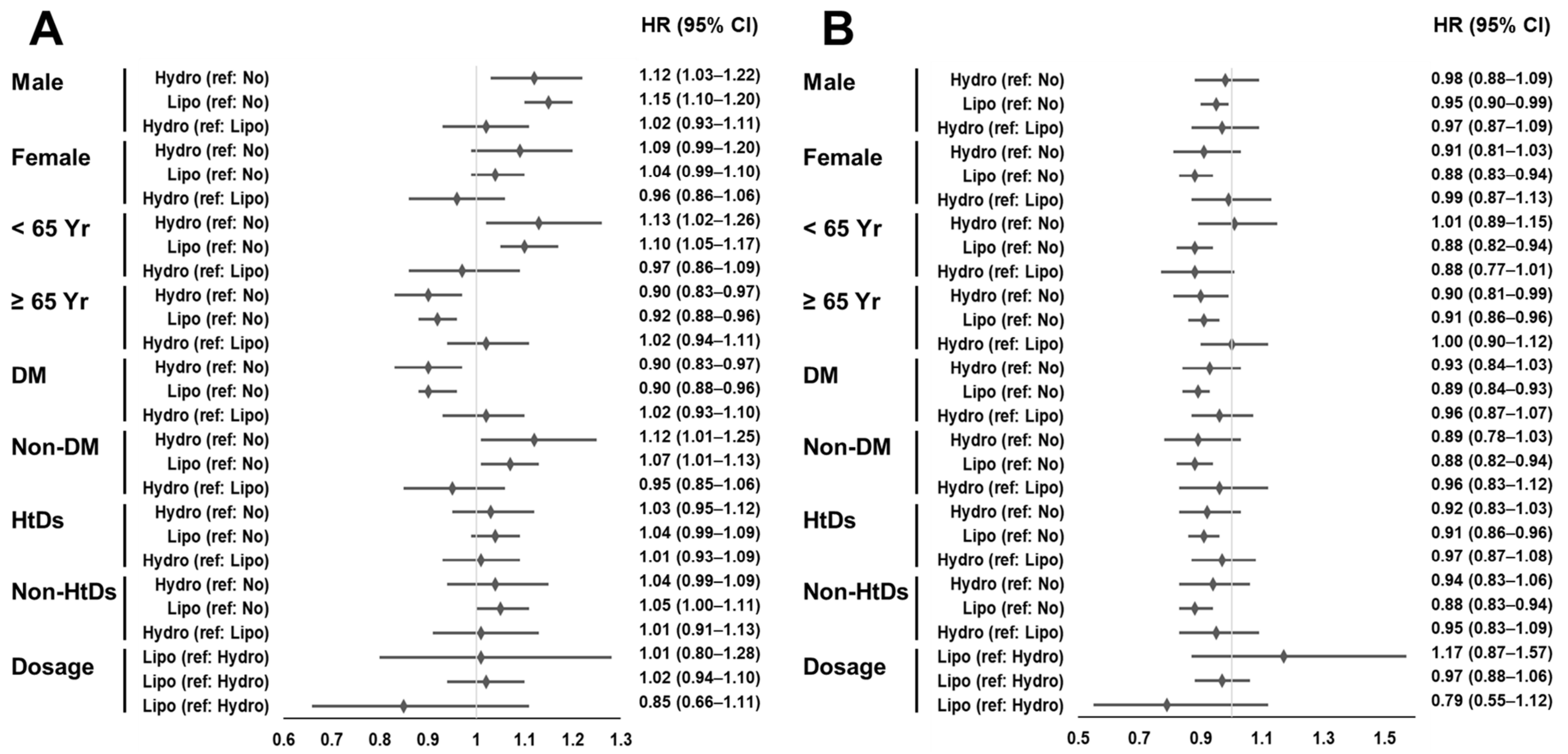

Figure S1. Forest plots of the HR and 95% CI according to subgroups

(A) Univariate Cox regression analysis (B) Multivariable Cox regression analyses. Adjustment according to age, sex, underlying cause of end-stage renal disease, Charlson comorbidity index score, type of vascular access, hemodialysis vintage, ultrafiltration volume, Kt/V_{urea}, hemoglobin, serum albumin, serum creatinine, serum phosphorus, serum calcium, systolic blood pressure, diastolic blood pressure, the use of

renin-angiotensin system blockers, clopidogrel, and aspirin, and myocardial infarction or congestive heart failure, and dosage of statin. In dosage category, reference was hydrophilic statin, and upper, middle, and low compartments reveal low, moderate, and high dosage subgroups.

Abbreviations: CI, confidence interval; DM, diabetes mellitus; No, patients without prescription of statins; Hydro, patients with prescription of hydrophilic statins; Lipo, patients with prescription of lipophilic statins; HR, hazard ratio; HtDs, patients with myocardial infarction or congestive heart failure; Non-HtDs, patients without myocardial infarction and congestive heart failure; Yr, years.
